# Trend in Devices and Digital Tools for Remote Consultation From Medical Providers to Specialists: Scoping Review

**DOI:** 10.2196/87559

**Published:** 2026-07-15

**Authors:** Risa Hara, Yosuke Hirakawa, Yuka Sugawara, Masao Iwagami, Ryota Inokuchi, Masaomi Nangaku

**Affiliations:** 1 Division of Nephrology and Endocrinology, Graduate School of Medicine, The University of Tokyo, 7-3-1 Hongo, Bunkyo-ku, Tokyo, 113-0085, Japan, 81 3-3815-5411, 81 3-5800-9826; 2 Department of Digital Health, Social Medicine Group, Institute of Medicine, University of Tsukuba, Tsukuba, Ibaraki, Japan; 3 Department of Clinical Engneering, The University of Tokyo Hospital, Bunkyo, Tokyo, Japan

**Keywords:** telemedicine, teleconsultation, remote consultation, medical consultation, doctor-to-doctor consultation, doctor-to-patient with doctor consultation, digital technology, mobile health

## Abstract

**Background:**

Digital health care technologies, including mobile applications and telemedicine platforms, have transformed how medical professionals communicate and deliver care. Remote consultation by doctors plays a vital role in ensuring access to appropriate expertise, particularly in medically underserved or geographically remote areas. However, the diversity in technological modalities, devices, and patterns of use across specialties and regions has not been systematically mapped.

**Objective:**

This study aimed to explore the current status and characteristics of teleconsultations among medical providers and specialists, focusing on device use, consultation modalities, clinical specialties, and regional differences. Through this approach, we aimed to provide a comprehensive overview of technological and practical trends in mobile health (mHealth) and telemedicine.

**Methods:**

A systematic scoping review was conducted in accordance with PRISMA-ScR (Preferred Reporting Items for Systematic Reviews and Meta-Analyses extension for Scoping Reviews) guidelines using the MEDLINE and Embase databases. The search covered studies published up to February 2026, with no restrictions on the publication year. Studies meeting the predefined inclusion criteria were also included.

**Results:**

A total of 255 citations were screened, and 99 articles were included. Studies were analyzed according to consultation method, target, and regional characteristics. Of these, 81 out of 99 (81.8%) articles were in the doctor-to-doctor category. Email, web, or app-based platforms were the most common. In addition, 86 out of 99 studies used medical images, most frequently photographs. Orthopedics and dermatology were the most frequently involved specialties, followed by internal medicine. Regarding the region, 60 of 99 studies were domestic, and the other 39 studies were international. Rural-to-urban domestic consultations comprised 27 out of 99 (27.3%) studies, whereas consultations from low- and middle-income countries and high-income countries accounted for 11 of 99 (11%) studies.

**Conclusions:**

This review examined doctor-to-doctor and doctor-to-patient consultations with doctor involvement. Specialties in which medical images are central, such as orthopedics and dermatology, were more frequently represented than in other fields. This highlights disparities in the use of teleconsultation across clinical disciplines and suggests that addressing these imbalances is essential for broader adoption. Furthermore, the findings indicated a progressive shift from videoconference-based interactions to mobile and app-based platforms, reflecting ongoing technological advancements. Optimizing the integration of these digital tools and promoting equitable access are critical for enhancing the quality and reach of teleconsultation practices in future digital health systems.

## Introduction

### Background

Teleconsultation is a form of telemedicine in which specialists and other health care professionals provide advice and clinical support to physicians and patients using information and communication technologies [1]. Such consultations play a crucial role in ensuring appropriate care, particularly when specialty departments are unavailable, or services are delivered in underserved or remote areas. Teleconsultation applies to diverse scenarios including routine outpatient care, emergency response, and multiple specialties. Their importance has grown since the COVID-19 pandemic, accelerating remote medical care and creating new health care delivery models [2]. Teleconsultation has demonstrated strong potential for bridging gaps in access where specialists are scarce.

Teleconsultation is commonly divided into two types: doctor-to-doctor consultations, in which physicians seek guidance from specialists, and doctor-to-patient consultations with doctor involvement, in which a physician mediates communication between the patient and specialist [3]. Implementation methods include telephone calls, email, and web or app-based platforms including store-and-forward or synchronous styles. Devices and platforms continue to evolve with technology, and the choice often depends on infrastructure, privacy requirements, communication environment, and clinical context [4].

In recent years, the rapid advancement in digital health technologies has diversified the implementation environment for telemedicine. These innovations have enabled rapid and detailed teleconsultation across geographic and institutional boundaries, expanding access to specialist expertise and transforming the conduct of remote collaboration in health care.

Against this backdrop, this study conducted a scoping review of the current status of teleconsultation to clarify the technological and practical trends in the evolving landscape of digital and remote medical communication.

### Objectives

This study aimed to clarify the technological trends and clinical characteristics in doctor-to-doctor and doctor-to-patient consultations involving doctors. We conducted a scoping review of doctor-to-doctor and doctor-to-patient consultations with doctor involvement. We comprehensively analyzed the implementation methods, devices used, and clinical scenarios, in order to clarify the challenges and opportunities for expanding the use of teleconsultation in the future.

## Methods

### Study Design

This scoping review was conducted in accordance with the PRISMA-ScR (Preferred Reporting Items for Systematic Reviews and Meta-Analyses extension for Scoping Reviews) guidelines, and the detailed methodology is provided in Checklist 1.

### Data Sources and Search Strategy

A systematic search was performed using the MEDLINE and Embase databases. The search covered studies published up to February 28, 2026, with no restrictions on the publication year. The search strategy was designed to capture studies reporting teleconsultations from doctors to doctors. Search terms included: MEDLINE: (telemedicine OR “online medical care” OR “teleconsultation” OR “online consultation” OR “telemedical consultation”) AND (“D to D” OR “doctor to doctor” OR “physician to physician”); Embase: (telemedicine OR “online medical care” OR teleconsultation OR “online consultation” OR “telemedical consultation”) AND (“D to D” OR “doctor to doctor” OR “physician to physician”). Details of the search strings are provided in Multimedia Appendix 1. Gray literature was excluded from this review. In addition, a single backward citation search was performed on all identified review articles to broaden coverage. We also conducted a robustness check using only the results of the database search to confirm that the findings were consistent with those of the overall analysis.

### Study Selection

After deduplication, titles and abstracts were screened using Covidence software (Veritas Health Innovation) by 2 independent reviewers (RH and YH). Full-text articles were then assessed using predefined inclusion and exclusion criteria. Studies were included if they reported teleconsultation between medical providers and specialists, regardless of whether patients were present. The exclusion criteria were as follows: (1) protocols and proposals, (2) systematic reviews, commentaries, and scoping reviews, (3) non-full-text publications, (4) publications published after February 2026, and (5) articles that were not published in English. Disagreements were resolved by consensus with a third reviewer (YS). The Cohen kappa coefficient was calculated to ensure the reliability of the study selection process. In this assessment, the kappa value was calculated using the following formula: kappa = (Po – Pe) / (1 – Pe), where Po represents the observed proportion of agreement, and Pe represents the hypothetical probability of chance agreement between the 2 independent reviewers (YH and RH). The results indicated substantial agreement between these 2 reviewers (YH and RH), with a kappa value of 0.603 for the full-text reviews. These results indicate substantial agreement between reviewers (YH and RH), suggesting that the screening process was conducted with a high degree of reliability. Discrepancies were resolved by a third reviewer.

### Data Extraction

Data extraction was conducted independently by two reviewers (RH and YH) using Covidence software (Veritas Health Innovation) and Excel (Microsoft Corp). Extracted data included: (1) citation details, (2) country of origin, (3) geographic characteristics (domestic or international, urban or rural), (4) medical specialties, (5) patient characteristics (adult or child), (6) evaluation criteria, (7) consultation methods (eg, email and telephone), (8) use of medical images, (9) inclusion of patients in consultations, and (10) reported costs. This review has been registered in the Open Science Framework Registry [5].

### Terminology and Classification Criteria

Due to variations in definitions, this study classified consultations as rural only when the original articles explicitly described the setting as geographically remote or lacking medical resources. Consultations conducted in remote areas were not considered rural unless these characteristics were clearly stated. Low- and middle-income countries (LMICs) and high-income countries (HICs) were classified according to World Bank classification. We categorized consultation methods by using web or app-based platforms into “Web or app-based synchronous platforms” for real-time engagement and “Web or app-based store-and-forward platforms” for asynchronous data transmission via integrated chat or email-based interfaces, because consultation modalities should be categorized into synchronous (real-time) and asynchronous (store-and-forward) interactions. In addition, when consultations were conducted synchronously via modalities such as videoconferencing, we changed the designation from “Store-and-forward video” (asynchronous) to “Real-time video” (synchronous) and performed the analysis again.

### Data Analysis

Fisher exact test was performed to evaluate the differences between domestic and international teleconsultations both in Table 1 and Table S2 in (Multimedia Appendix 2). A *P* value of ≤.05 was considered statistically significant. No adjustments were made for multiple comparisons; therefore, all statistical analyses were exploratory.

**Table 1.** Methods of consultation by domestic or international consultation. Some studies used multiple devices or modalities; therefore, the total number of modality occurrences exceeded that of the number of included studies (n=99) because some studies used multiple modalities. Counts reflect individual modality occurrences rather than unique studies.

Characteristics	Domestic consultations (n=60)	International consultations (n=39)	*P* value
Web or app-based synchronous platforms	25 (40.3)	15 (34.9)	.69
Email	15 (24.2)	19 (44.2)	.04^a^
Web or app-based store-and-forward platforms	11 (17.7)	6 (14)	.78
Phone	6 (9.7)	1 (2.3)	.24
Other	4 (6.5)	1 (2.3)	.65
Unknown	1 (1.6)	1 (2.3)	≥.99

a *P*<.05.

### Review Period

The review process was conducted between March 2024 and March 2026.

### Ethical Considerations

This study is a scoping review of previously published literature. Ethical approval was not required because no human participants or identifiable personal data were involved.

## Results

### Literature Search

**Figure 1.**
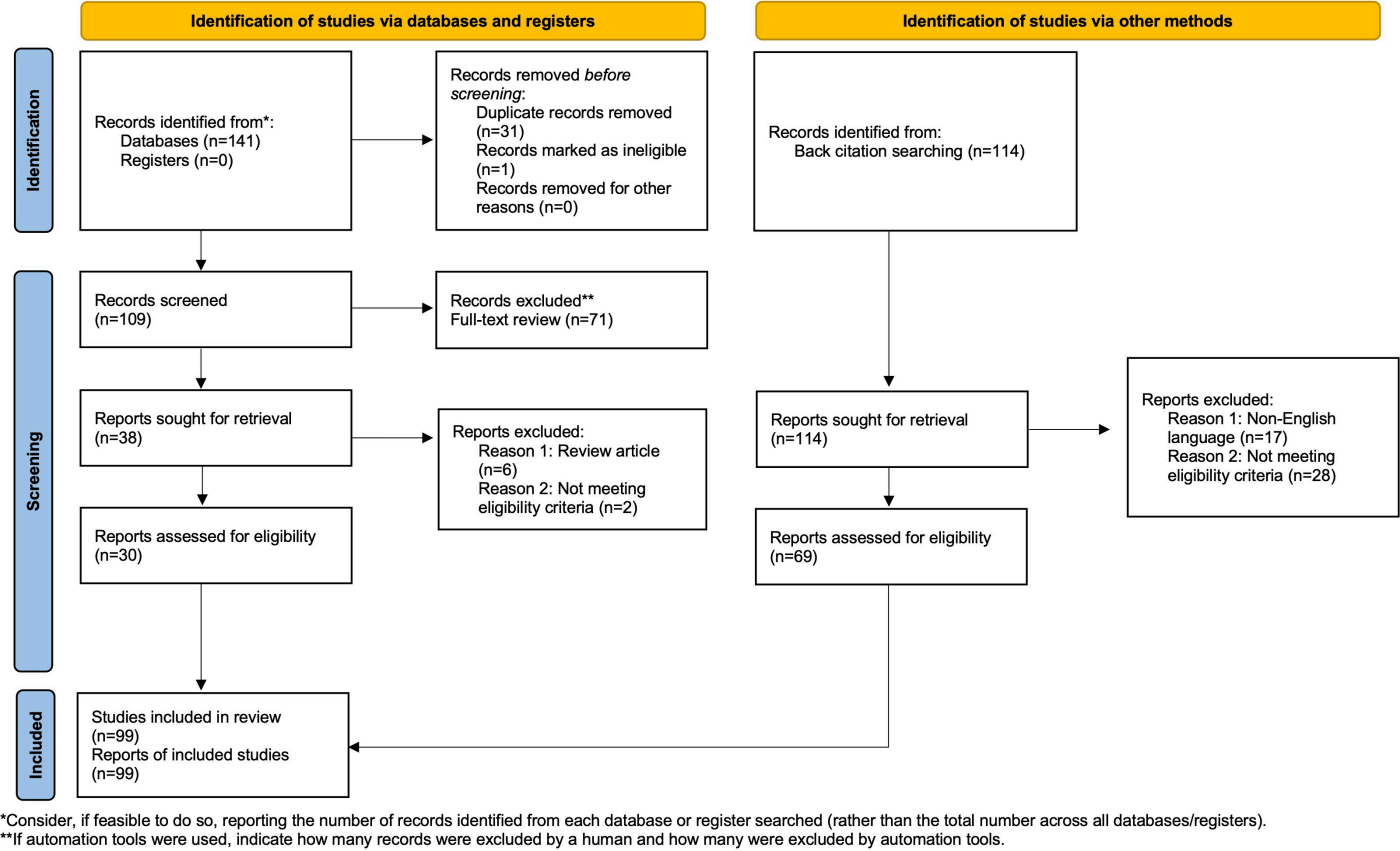
PRISMA (Preferred Reporting Items for Systematic Reviews and Meta-Analyses) 2020 flow diagram for new systematic reviews which included searches of databases, registers, and other sources.

Initially, 141 articles were identified through database searches. After deduplication, 109 records were selected for screening. The screening of title and abstract reduced the number of potentially eligible articles to 38. Among these, 6 review articles and 2 articles were excluded after a full-text review, leaving 30 studies. A backward citation search of the 6 review articles identified during the full-text review yielded an additional 114 articles for full-text review. Among these, 28 were excluded based on the eligibility criteria, and 17 were excluded for publications in non-English languages. Finally, a further 69 articles were included. Overall, 99 studies [6-104] (Multimedia Appendix 3) were included in the qualitative analysis. A PRISMA-ScR flow diagram summarizing the study selection process is shown in Figure 1. A list of all 99 studies and their information is provided in Multimedia Appendix 3. After excluding backward citation studies, the total number of included studies was 30 [9-18,20-25,86-97,104].

### Evaluation Items of the Included Studies

Following the study selection process, we extracted and categorized key entities from each included article based on the following criteria: (1) citation details (authors and year of publication); (2) country of origin; (3) geographic characteristics, including whether the study was domestic or international and whether it was conducted in urban or rural settings; (4) target medical departments or organs; (5) characteristics of the target population, including sample size and whether participants were adults or children; (6) evaluation criteria used in each study (such as clinical outcomes, user satisfaction, or feasibility); (7) consultation method, including email, telephone call, videoconferencing, or app-based platforms; (8) whether medical images were used, and if so, what types of images were used (eg, photographs and radiological images); (9) whether the consultation was conducted exclusively among health care professionals or whether patients were also involved; and (10) cost reporting and financial aspects, if these were referenced in the study.

In cases in which specific data were unavailable in the original publication, the information was reported as missing. These extracted data points formed the basis for the categorization and analysis presented in the subsequent sections.

### Study Description

The characteristics of the studies are shown in Table 2. All 99 articles were published between 1996 and 2026. In particular, the COVID-19 pandemic in 2020 accelerated the global adoption of medical practices designed to minimize face-to-face interactions through telemedicine. The countries in which the articles were published are indicated by region in Table 2. North America accounted for the largest number of articles (30/99, 30.3% studies 9,12,15,18,21,23,25,27,31,33,35,47,49-53,55-57,69,74-79,93,101,103]), followed by Europe (17 studies [10,20,22,29,42,44-46,48,54,58,62,65,70,72,85,87], 17.2%). Overall, 19 (19.2%) studies [14,19,39-41,43,60,63,64,71,73,81-84,91,92,97,104] reported on cross-national or international consultations. Owing to the nature of the telemedicine field, 3 (3%) studies [7,67,68] were conducted in Antarctica, which lacks medical facilities and staff. The analysis, excluding studies identified through backward citations, was generally consistent with the main results (Table S1 in Multimedia Appendix 2).

**Table 2.** Study characteristics. Percentages are rounded to one decimal place; component sums may not equal the total. The total number of modality occurrences and target medical departments exceeded the number of included studies (n=99) because some studies included multiple consultation characteristics. Counts reflect individual modality occurrences rather than unique studies.

Characteristic	Studies, n (%)
Publication year	
1996‐2000	3 (3)
2001‐2005	5 (5.1)
2006‐2010	15 (15.2)
2011‐2015	34 (34.3)
2016‐2020	17 (17.2)
2021‐2025	24 (24.2)
2026	1 (1)
Geographic area of teleconsultation activity	
North America	30 (30.3)
South America	2 (2)
Asia	15 (15.2)
Europe	17 (17.2)
Middle East	4 (4)
Africa	4 (4)
Oceania	5 (5.1)
Antarctica	3 (3)
International	19 (19.2)
Target medical department	
Orthopedics	22 (21.2)
Dermatology	19 (18.3)
Internal medicine	15 (14.4)
Neurology	5 (4.8)
Cardiology	3 (2.9)
Infectiology	3 (2.9)
Oncology	1 (1)
Pulmonology	1 (1)
Hepatology	1 (1)
Pain medicine	1 (1)
Surgery	7 (6.7)
Emergency room	6 (5.8)
Ophthalmology	6 (5.8)
Pediatrics	4 (3.8)
Obstetrics	1 (1)
Oral surgery	1 (1)
Psychiatry	1 (1)
Pathology	1 (1)
Radiology	1 (1)
Not specified or multidisciplinary	20 (19.2)
Consultation direction	
Primary care provider to specialist	62 (62.6)
Specialist to specialist	36 (36.4)
Both of the above	1 (1)
Patient involvement	
Doctor to doctor	81 (81.8)
Doctor to patient consultation with doctor	14 (14.1)
Both of the above	4 (4)
Domestic or international consultations	
Domestic consultations	60 (60.6)
Rural to urban area	27 (27.3)
Not specified	33 (33.3)
International consultations	39 (39.4)
LMICs^a^ to HICs^b^ area	11 (11.1)
Military installations	10 (10.1)
Antarctica	3 (3)
Not specified	15 (15.2)

a LMICs: low- and middle-income countries

b HICs: high-income countries.

### Domestic or International Consultation

Of the 99 included articles, 60 (60.6%) studies [6,8,10-13,15-18,20-27,29,30,32-36,39,41-58,62,85-88,93-96,98-103] reported domestic consultations, and the remaining 39 (39.4%) studies [7,14,19,28,31,37,38,40,59-61,63-84,89-92,97,104] reported international consultations (Table 2). Among domestic cases, 27 studies [11,12,14-18,21,26,28-33,37,55,58,62,85-87,92,94-96] described communication from rural to urban areas, particularly consultations from rural clinics to urban hospitals. Consultations from LMICs to HICs accounted for 11 of the international cases, reflecting the role of teleconsultation in addressing global disparities in access to specialist care.

### Target Medical Departments

As summarized in Table 2, the included studies covered a wide range of medical specialties. Among the 99 studies, the most frequently targeted departments were orthopedics (22, 21.2% studies [29-46,54,79,97,100]) and dermatology (19, 18.3% studies [12,15,17,25-28,47-53,66,82,83,85,102]). Internal medicine was the third most frequently reported field (15, 14.4% studies [6,7,9,13,22-24,58,60,62,63,73,77,78,97]). Within this field, neurology was the most common subspecialty, followed by cardiology (3, 2.9% studies [7,62,77]) and infectious diseases (3, 2.9% studies [13,73,78]). Neurology consultations are often concerned with emergency transport cases such as cerebral hemorrhage and stroke [6], and specialist consultations for conditions such as headache and tremor [105]. In contrast, cardiology consultations are frequently conducted for urgent conditions, including pericardial effusion secondary to pericarditis, often using tele-echocardiographic evaluation [7], and commonly involve teleconsultations based on readily shareable diagnostic data, such as electrocardiograms and chest radiographs [8].

In addition, consultations were reported in surgery (7, 6.7% studies [54,55,59,61,64,70,97]), ophthalmology (6, 5.8% studies [11,20,76,81,93,96]), pediatrics (4, 3.8% studies [38,65,87,101]), including pediatric surgery, pediatric orthopedics, and pediatric oncology, and emergency medicine (6, 5.8% studies [18,56,57,71,74,98]). One article each reported on pathological and psychiatric consultations. The remaining 20 studies [8,10,14,16,19,67-69,72,75,84,88-92,95,99,103,104] primarily addressed the technical aspects of teleconsultation or discussed teleconsultation without specifying the disease area.

### Consultation Direction and Patient Involvement

The directional flow and structural forms of teleconsultation varied across the included studies. We categorized the consultations into two primary types based on direction: Of all 99 studies, primary care provider-to-specialist and specialist-to-specialist. Most studies (62/99, 62.6% studies [6-19,21,23-27,29-31,33,34,37,40,41,47-51,53-55,57,61,67-69,72,74-79,84,85,87,89,92,93,95-99,101,103,104]) described primary care provider–to–specialist consultations in which general practitioners or community-based physicians sought expert input for diagnostic clarification or treatment decisions. These interactions often support primary care providers in underserved rural regions.

In contrast, 36 of 99 (36.4%) studies [10,20,22,28,32,35,36,38,39,42-46,52,56,58-60,62-66,70,71,73,80-83,86,88,94,100,102] reported specialist-to-specialist consultations, in which subspecialists communicated with other specialists; for example, a general surgeon consulted a radiologist or dermatologist. One (1%) study [10] described both consultation styles.

Regarding the structural form of consultations, we also evaluated for direct patient involvement. A substantial proportion of the studies (81/99, 81.8% studies [6,9,10,12-23,25-31,33,35,36,38-42,47-55,57-70,72-79,81-85,89-92,94-104]) involved doctor-to-doctor interactions conducted exclusively among health care professionals without direct patient participation. These models typically entail the exchange of clinical data, imaging data, or summary reports via electronic platforms.

Meanwhile, 14 of 99 (14.1%) studies [7,8,11,24,32,34,37,43-46,56,71,80] featured doctor-to-patient with-doctor models, in which a physician facilitated communication between the patient and a remote specialist. Such arrangements are often used in geographically isolated or low-resource settings where direct specialist access is unavailable.

When comparing doctor-to-doctor and doctor-to-patient consultations with doctor involvement, domestic cases were predominantly conducted as doctor-to-doctor consultations, whereas international cases were markedly more frequent in the doctor-to-patient with doctor format. North America showed a significantly higher proportion of doctor-to-doctor consultations, whereas Oceania had significantly more doctor-to-patient consultations involving doctors. Videoconferencing was significantly more frequent in doctor-to-patient consultations with doctor involvement, whereas email, although not statistically significant, tended to be more common in doctor–to–doctor consultations. In terms of imaging modalities, photographs were significantly more frequent in doctor-to-doctor consultations, whereas videos were significantly more common in doctor-to-patient consultations with doctor involvement. No significant differences were observed for other imaging modalities; however, no cases in the doctor-to-patient with doctor group lacked imaging usage. Among the clinical specialties, orthopedics was significantly more frequent in doctor-to-patient consultations with doctor involvement, whereas dermatology was significantly more common in doctor-to-doctor consultations, with no significant differences observed for other specialties.

### Methods of Consultation

All the articles were classified according to the methods used for teleconsultation, including email, telephone, web or app-based synchronous platform, web or app-based store-and-forward platform, other methods, and articles in which the method could not be identified.

**Figure 2.**
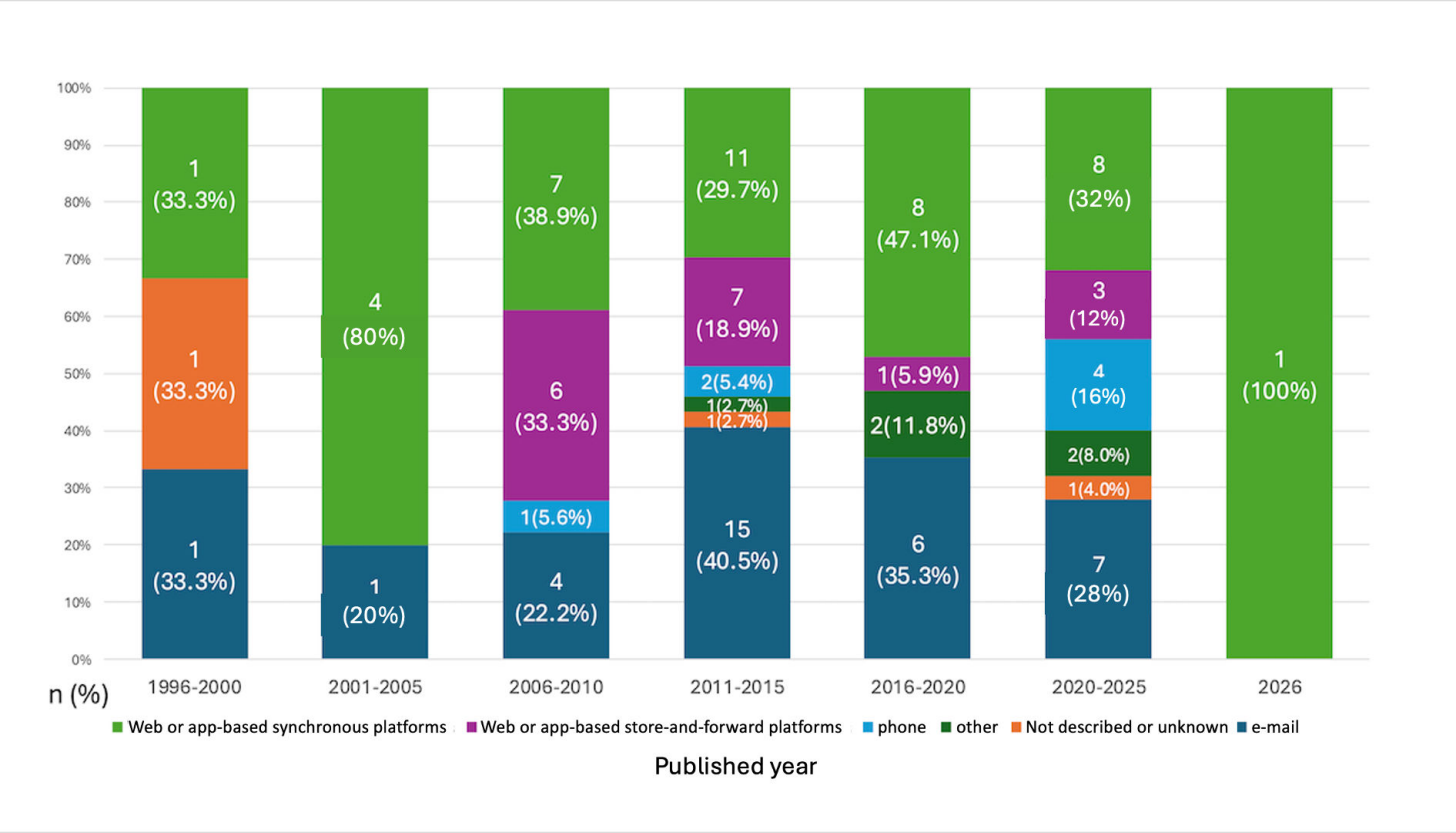
Time trends of in consultation methods; consultation methods across five-year periods from 1996 to 2026.

Figure 2 presents a color-coded vertical bar chart of the consultation methods for the five-year period from 1996 to 2026. Figure S2 in Multimedia Appendix 4 also presents the same bar chart in 30 articles, excluding backward-searching studies. Email was used consistently since 2001, and a significant shift toward web or app-based synchronous platforms was observed in more recent years. Web or app-based platforms include independently developed web services within specific countries or hospital groups, reflecting the broader global adoption of such digital interfaces.

The differences in teleconsultation methods between domestic and international consultations are presented in Table 1 and Table S2 (Multimedia Appendix 2) with and without backward citation studies, respectively. Email was significantly more common in international consultations than in domestic consultations (*P*=.04), while when backward citation studies were excluded, the difference was not statistically significant. Reflecting the unit of analysis as modality occurrences, the total count exceeded the number of included studies because some programs used multiple methods.

### Image Modalities

Of the 99 studies reviewed, 86 (86.9%) [6-9,11,12,15-18,20,24-73,75-88,90-94,96-98,100-102] reported the use of some form of medical imaging during consultations. Among these 86 studies, clinical photographs (n=53, 31.9% [11,12,15,17,18,25-28,36-38,40,41,47-53,59,61,63,64,66,67,69-73,75-79,81-85,88,90,92-94,96,98,100-102]), real-time video (n=36, 21.7% [6-8,11,24,29-31,34-37,39,43-46,54-60,62,64,65,69,71,80,86-88,90-92]), and radiographic imaging (n=44, 36.5% [6,7,9,16,29,30,32-34,36-38,40-46,54,59,61,62,72,75,79-81,96,97,100]) were the most common modalities, followed by magnetic resonance imaging, specialized imaging, ultrasonography, physiological data, store-and-forward video, pictures, and pathological imaging. As some studies used multiple modalities, the total number of identified modality occurrences was 166. The details are summarized in Table 3. Radiographic imaging included radiography, computed tomography, and angiography, whereas specialized imaging included dermoscopic images in dermatology and eye fundus photographs in ophthalmology. Also, physiological data included electrocardiography, pulmonary function tests, and cardiotocography, and pictures included pictures drawn by patients and burn diagrams. Analyses excluding studies identified through backward citation yielded generally consistent results (Table S3 in Multimedia Appendix 2).

**Table 3.** Technical and implementation features. Some studies used multiple devices or modalities; therefore, the total number of modality occurrences exceeded that of the number of included studies (n=99) because some studies used multiple modalities. Counts reflect individual modality occurrences rather than unique studies.

Image modalities use	Studies, n (%)
Yes	86 (86.9)
Photograph	53 (31.9)
Radiographic imaging	44 (26.5)
Real-time video	36 (21.7)
Specialized imaging	8 (4.8)
Ultrasound imaging	7 (4.2)
MRI^a^	6 (3.6)
Physiological data	5 (3)
Pathological imaging	3 (1.8)
Picture	3 (1.8)
Store-and-forward video	1 (0.6)
Unknown, no description provided	9 (9.1)
None	4 (4)

a MRI: magnetic resonance imaging.

The most frequently used modality, clinical photography, was used in 53 studies [11,12,15,17,18,25-28,36-38,40,41,47-53,59,61,63,64,66,67,69-73,75-79,81-85,88,90,92-94,96,98,100-102], particularly in dermatology and orthopedic surgery. Photography allows for rapid and clear communication of visible physical symptoms such as rashes, wounds, joint deformities, and skin lesions. With the advent of high-resolution cameras and secure image-sharing applications, teleconsultation has been increasingly conducted using high-quality images in a simplified and privacy-conscious manner. The remaining 4 of 99 (4%) studies [13,14,21,99] did not use any medical images during their consultation process, and 9 (9.1%) studies [10,19,22,23,74,89,95,103,104] provided no description of the modalities used.

## Discussion

### Principal Results

This scoping review synthesized 99 studies (published until February 2026) to provide an overview of current teleconsultation practices across clinical settings, regions, and modalities. We observed substantial variations in implementation according to specialty, consultation method, and geographical context. Within the context of doctor-to-doctor consultations, this pattern suggests that image-intensive specialties may be more compatible with remote consultation formats; therefore, these models were adopted more readily.

### Technological Evolution and Communication Modalities

Our review identified a gradual shift from telephone to email and web or app-based platforms (both store-and-forward or synchronous type), reflecting broader advances in the telemedicine infrastructure [106]. These platforms enable seamless transmission of images and clinical data, thereby enhancing the quality of remote evaluation, and are especially common in international consultations, as their asynchronous format overcomes time-zone barriers.

Web or app-based platforms offering structured input and secure image sharing are well-suited to image-dependent specialties [107]. With the widespread use of mobile devices and growth of mobile health (mHealth) platforms, teleconsultations have become increasingly accessible to health care professionals, even in resource-limited environments. These trends indicate that technology accessibility and platform functionality are central to teleconsultation.

### Image Modalities in Teleconsultation

A notable finding of this review is the high frequency of medical imaging use in teleconsultations, particularly in clinical photographs and radiological imaging. These images are not only common but also integral to the consultation process. Among the included studies, the majority used some form of visual data to support diagnosis or decision-making. Photographs of skin lesions, wounds, or orthopedic conditions are frequently transmitted via email or app-based platforms, enabling remote specialists to provide accurate advice without direct patient contact. Radiological images, including x-ray and computed tomography scans, have been used in many consultations, particularly in orthopedics and emergency care.

Recently, the potential use of 3D imaging technology for teleconsultation and telementoring in oral and maxillofacial surgeries has been investigated [106]. Compared to traditional 2D images, 3D images and virtual-reality applications created from 3D data can enable clinicians to understand complex anatomical structures more clearly and share surgical plans more effectively. At present, creating 3D data requires expensive equipment and software, as well as a fast and stable Internet connection for data transfer. However, this technology has already been adopted to support communication and education among doctors in remote areas [108][109]. In addition, mobile-based image capturing and AI-supported image analysis tools have been increasingly integrated into teleconsultation workflows across multiple clinical specialties, including ophthalmology [110]. Collectively, these developments highlight the critical role of visual diagnostic tools in remote clinical settings. Improving the accessibility of image capturing and sharing, particularly in resource-limited settings, can expand teleconsultation to a broader range of clinical scenarios.

### Clinical Specialties and Image-Driven Practices

We found a significant disparity in teleconsultation activities across clinical departments. Medical fields such as orthopedics and dermatology accounted for a substantial proportion of the included studies. These specialties frequently rely on visual cues, and clinical decisions are often made based on images alone, particularly clinical photographs or radiological images. This pattern suggests that image-intensive specialties are more compatible with remote consultation formats and have, therefore, adopted teleconsultation more readily. In a robustness check excluding studies identified through backward citations, no significant differences were observed across specialties (Table S1 in Multimedia Appendix 2), whereas the proportion of orthopedics was somewhat lower, suggesting a potential influence of citation-based inclusion. Fields such as internal medicine are relatively underrepresented compared with orthopedics and dermatology. This may be because these specialties often require more integrative assessments involving history-taking, laboratory data, and physical examinations, which are difficult to fully replicate in a remote setting. Several studies have suggested that the adoption of telemedicine varies across medical specialties and clinical conditions, largely due to differences in the need for physical examinations [111]. The increasing use of mobile communication tools and wearable sensors may help expand teleconsultation to nonimage-based specialties by enabling the real-time transmission of physiological or biometric data.

### The Difference Between General Telemedicine and Teleconsultation

As general telemedicine and the types of teleconsultations examined in this study have different goals and evaluation criteria, the mix of specialties involved and reasons for adoption are not the same.

In the United States, since the COVID-19 pandemic, telemedicine has been used more frequently in history-focused fields such as psychiatry, endocrinology (particularly diabetes care), and neurology [112]. It has been widely used in pediatrics and gynecology. One reason is that childcare responsibilities and work demands often make clinic visits difficult. Telemedicine reduces travel and time burdens, thereby enabling more visits to be completed [113]. Telemedicine delivers care directly to patients in real time and is typically evaluated based on visit completion rates, reduced missed or interrupted visits, and patient satisfaction [114].

In contrast, teleconsultation aims to obtain specialist advice to improve diagnostic confidence, shorten patient wait times, support primary care decision-making, and, in some cases, avoid in-person referrals or transfers. Teleconsultation is particularly common in specialties that rely heavily on imaging, such as dermatology and orthopedics.

### Opportunities and Remaining Challenges

Several challenges must be addressed to support the continued expansion of teleconsultation. First, underrepresented specialties require greater integration. Even in fields where image-based diagnostics are uncommon, transmitting laboratory results or PDF reports can help bridge this gap. Devices and platforms should be designed with flexibility and interoperability to accommodate diverse clinical data formats [4].

Recent studies in nephrology have illustrated both the obstacles and potential solutions for nonimage-intensive specialties. Our previous study [115] noted that telemedicine in nephrology is underused because of difficulties in conducting physical examinations, laboratory tests, and remote imaging. Promising approaches include estimating serum creatinine from saliva or tear fluid, measuring hemoglobin via smartphone photographs of the conjunctiva or fingernails, and using motion-capture–assisted kidney ultrasound outside hospital settings. These innovations can be adapted more broadly within the field of internal medicine to enhance remote diagnostics.

This review also identified 29 studies [8,9,12-14,18,19,30,33,34,41,43,44,47,54,59,69,77,79,82,87-89,92,93,95,96,101,104] referencing cost-related benefits, such as reduced patient transport and faster diagnostic decisions [9]. These findings highlight the economic advantages, although more systematic cost-effectiveness analyses are needed.

Finally, as teleconsultation expands globally, disparities in reimbursement models and regulations present barriers [116]. Although countries such as the United States have established broader billing systems, such as current procedural terminology (CPT) codes for interprofessional eConsults, others, including Japan, still face implementation challenges. Although Japan has integrated telemedicine into its Medical Care Act and has established specific medical fees for certain tele-diagnostic services; nonetheless, the reimbursement rates for most physician-to-physician consultations remain relatively low, and eligibility criteria are often too restrictive to support diverse interprofessional interactions across clinical specialties, which results in a more fragmented system than those with standardized, cross-specialty billing mechanisms. The development of coordinated international frameworks and standardized protocols is required for equitable and sustainable implementation [117].

### Limitations

Although our scoping review was thorough, it had certain limitations that should be acknowledged. First, we searched the MEDLINE and Embase databases and included peer-reviewed full-text articles published in English. Therefore, studies published in other languages and the gray literature were not included, which may have reduced the comprehensiveness of the review. Specifically, this search strategy may have underrepresented teleconsultation initiatives in low-resource settings and international programs, where activities are more frequently documented in regional languages or unpublished institutional reports. Consequently, the global trends identified in this review should be interpreted as being primarily reflective of peer-reviewed, English-language literature rather than of a truly exhaustive global record. Second, a backward citation search was conducted to supplement the database search. This method may have introduced a selection bias by favoring studies that were more frequently cited or belonged to similar research networks, thereby affecting the overall balance of the study results. We conducted the same analyses, excluding studies identified through backward citation and confirmed that most of the evaluated characteristics showed no significant differences, whereas significant differences were observed in publication year (*P*<.001) and consultation direction (*P*=.02), owing to the retrieval of older literature and specific consultation types through backward searching (Tables S1-S3 in Multimedia Appendix 2). Third, narrative synthesis is a secondary analysis of data that focuses on the interpretations presented by the authors of original papers and is not based on primary data. Consequently, our findings represent an interpretation of the evidence and should be viewed as a heuristic framework, rather than as a definitive conclusion. Finally, this scoping review may reflect published research on teleconsultation rather than the full scope of real-world implementation because several telemedicine practices and the latest technological or regulatory shifts may not be formally documented in the literature or may be missed owing to the inherent time lag between practical innovation and academic publication. As such, the specialty distributions observed here could be influenced by research activity and publication patterns (including backward snowballing) as much as, or perhaps more than, the actual service uptake.

### Conclusions

This scoping review highlights the diverse practices and evolving nature of teleconsultation, with particular emphasis on doctor-to-doctor and doctor-to-patient-with-doctor models. We found that image-based specialties such as orthopedics and dermatology are leading the implementation of teleconsultation, supported by the increasing use of image-compatible platforms such as email and mobile applications. The integration of visual data plays a crucial role in enabling remote clinical decision-making.

Despite these advancements, significant disparities remain across clinical fields and expanding teleconsultation into less image-dependent specialties will require innovations in data sharing and system design. Future progress in teleconsultation should focus on improving interoperability and integrating mobile technologies to promote equal access across regions and clinical areas. Continued innovation in digital health and supportive policies is essential for establishing teleconsultations as a stable and essential part of future health care systems.

## Supplementary material

10.2196/87559 Multimedia Appendix 1 Details of the search strings of this scoping review.

10.2196/87559 Multimedia Appendix 2 Results of analyses excluding studies identified through backward citation searches.

10.2196/87559 Multimedia Appendix 3 The full list of the 99 included studies and their primary characteristics.

10.2196/87559 Multimedia Appendix 4 Time trend of consultation methods; Consultation methods by five-year period from 2006-2026 without backward citation.

10.2196/87559 Checklist 1 PRISMA-ScR Checklist.

## Abbreviations

CPT: current procedural terminology
HIC: high-income country
LMIC: low- and middle-income country
mHealth: mobile health
PRISMA-ScR: Preferred Reporting Items for Systematic Reviews and Meta-Analyses extension for Scoping Reviews
