# Supplementary material for: Trend in Devices and Digital Tools for Remote Consultation From Medical Providers to Specialists: Scoping Review
Source: Online J Public Health Inform. 2026 Jul 15;18:e87559. doi: 10.2196/87559 (PMC13372300; doi:10.2196/87559)
Supplement: Multimedia Appendix 1 [file ojphi-v18-e87559-s001.pdf]

MEDLINE: (telemedicine OR "online medical care" OR teleconsultation OR "online consultation" OR "telemedical consultation") AND ("D to D" OR "doctor to doctor" OR "physician to physician")

Embase: ((telemedicine or "online medical care" or teleconsultation or "online consultation" or "telemedical consultation") and ("D to D" or "doctor to doctor" or "physician to physician")).mp. [mp=title, abstract, heading word, original title, device manufacturer, device trade name, keyword heading word, floating subheading word, candidate term word]
