# Supplementary material for: Trend in Devices and Digital Tools for Remote Consultation From Medical Providers to Specialists: Scoping Review
Source: Online J Public Health Inform. 2026 Jul 15;18:e87559. doi: 10.2196/87559 (PMC13372300; doi:10.2196/87559)
Supplement: Multimedia Appendix 2 [file ojphi-v18-e87559-s002.docx]

**Table S1.** Study characteristics

|  | Characteristic | n (%) |
| --- | --- | --- |
| Publication year | | |
|  | 1996–2000 | 0 (0) |
|  | 2001–2005 | 0 (0) |
|  | 2006–2010 | 2 (6.7) |
|  | 2011–2015 | 3 (10) |
|  | 2016–2020 | 4 (13.3) |
|  | 2021–2025 | 20 (66.7) |
|  | 2026 | 1 (3.3) |
| Geographic area of teleconsultation activity | | |
|  | North America | 8 (26.7) |
|  | South America | 1 (3.3) |
|  | Asia | 8 (26.7) |
|  | Europe | 4 (13.3) |
|  | Middle East | 2 (6.7) |
|  | Africa | 0 (0) |
|  | Oceania | 2 (6.7) |
|  | Antarctica | 0 (0) |
|  | International | 5 (16.7) |
| Target medical department | | |
|  | Orthopedics | 1 (2.9) |
|  | Dermatology | 4 (11.8) |
|  | Internal medicine | 6 (17.6) |
|  | Neurology | 2 (5.9) |
|  | Cardiology | 0 (0) |
|  | Infectiology | 1 (2.9) |
|  | Oncology | 0 (0) |
|  | Pulmonology | 1 (2.9) |
|  | Hepatology | 1 (2.9) |
|  | Pain medicine | 1 (2.9) |
|  | Surgery | 2 (5.9) |
|  | Emergency room | 1 (2.9) |
|  | Ophthalmology | 4 (11.8) |
|  | Obstetrics | 1 (2.9) |
|  | Oral surgery | 1 (2.9) |
|  | Pediatrics | 1 (2.9) |
|  | Pathology | 1 (2.9) |
|  | Psychiatry | 1 (2.9) |
|  | Radiology | 1 (2.9) |
|  | Not specified or multidisciplinary | 11 (32.4) |
| Consultation Direction | | |
|  | Primary care provider to specialist | 22 (73.3) |
|  | Specialist to specialist | 5 (16.7) |
|  | Both of the above | 1 (3.3) |
|  | Not specified | 2 (6.7) |
| Patient involvement | | |
|  | Doctor to doctor | 23 (76.7) |
|  | Doctor to patient consultation with doctor | 3 (10) |
|  | Both of the above | 4 (13.3) |
| Domestic or international consultations | | |
|  | Domestic consultations | 23 (76.7) |
|  | Rural to urban area | 8 (34.8) |
|  | Not specified | 15 (65.2) |
|  | International consultations | 7 (23.3) |
|  | LMICs to HICs area | 2 (28.6) |
|  | Military installations | 0 (0) |
|  | Antarctica | 0 (0) |
|  | Not specified | 5 (71.4) |

Percentages are rounded to one decimal place; component sums may not equal the total. Some studies used multiple devices or modalities; therefore, the total number of modality occurrences exceeded that of the number of included studies (n=30) because some studies employed multiple modalities. Counts reflect individual modality occurrences rather than unique studies.

**Table S2.** Methods of consultation by domestic or international consultation

|  | Domestic consultations  N=23 | International consultations  N=7 | P-value |
| --- | --- | --- | --- |
| Web or app-based synchronous platforms | 8 (32) | 5 (55.6) | .25 |
| E-mail | 6 (24) | 1 (11.1) | .65 |
| Web or app-based store-and-forward　platforms | 4 (16) | 1 (11.1) | ≥.99 |
| Phone | 4 (16) | 1 (11.1) | ≥.99 |
| Other | 2 (8) | 0 (0) | ≥.99 |
| Unknown | 1 (4) | 1 (11.1) | .46 |

Some studies used multiple devices or modalities; therefore, the total number of modality occurrences exceeded that of the number of included studies (n=30) because some studies employed multiple modalities. Counts reflect individual modality occurrences rather than unique studies.

Table S3. Technical and implementation features

| Image modalities use | |
| --- | --- |
| Yes | 47 (79.7) |
| Photograph | 13 (22) |
| Radiographic imaging | 16 (27.1) |
| Real-time video | 9 (15.3) |
| Specialized imaging | 5 (8.5) |
| Ultrasound imaging | 3 (5.1) |
| MRI | 2 (3.4) |
| Physiological data | 2 (3.4) |
| Pathological imaging | 2 (2) |
| Picture | 0 (0) |
| Store-and-forward video | 0 (0) |
| Unknown, no description provided | 6 (10.2) |
| None | 3 (5.1) |

Some studies used multiple devices or modalities; therefore, the total number of modality occurrences exceeded that of the number of included studies (n=30) because some studies employed multiple modalities. Counts reflect individual modality occurrences rather than unique studies.
