# Supplementary figures and images for: Trend in Devices and Digital Tools for Remote Consultation From Medical Providers to Specialists: Scoping Review
Source: Online J Public Health Inform. 2026 Jul 15;18:e87559. doi: 10.2196/87559 (PMC13372300; doi:10.2196/87559)

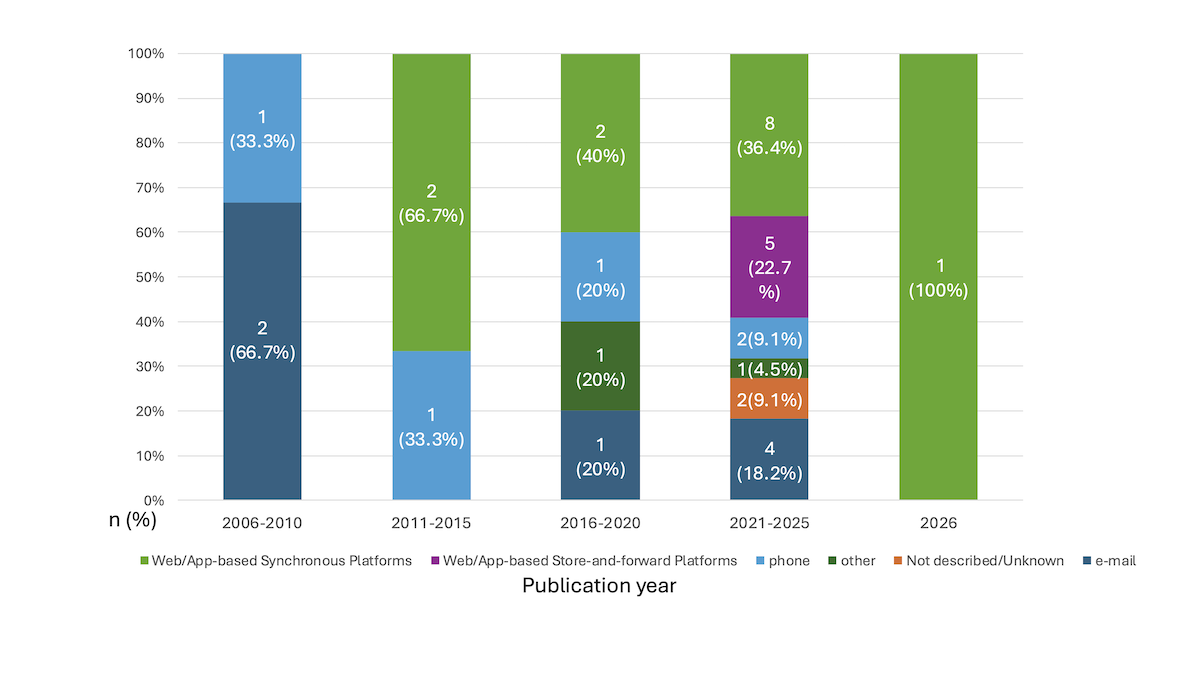

Supplement: Multimedia Appendix 4 [file ojphi-v18-e87559-s004.png]
